# Trends in health workers’ compliance with outpatient malaria case-management guidelines across malaria epidemiological zones in Kenya, 2010–2016

**DOI:** 10.1186/s12936-020-03479-z

**Published:** 2020-11-11

**Authors:** Beatrice Amboko, Kasia Stepniewska, Peter M. Macharia, Beatrice Machini, Philip Bejon, Robert W. Snow, Dejan Zurovac

**Affiliations:** 1 KEMRI-Wellcome Trust Research Programme, P.O. Box 43640-00100, Nairobi, Kenya; 2 WorldWide Antimalarial Resistance Network, Oxford, UK; 3 Centre for Tropical Medicine and Global Health, University of Oxford, Oxford, UK; 4 Division of National Malaria Programme, Ministry of Health, Nairobi, Kenya

**Keywords:** Malaria, Outpatient, Case-management, Malaria endemicity, Compliance, ‘Test and treat’, Kenya

## Abstract

**Background:**

Health workers' compliance with outpatient malaria case-management guidelines has been improving, specifically regarding the universal testing of suspected cases and the use of artemisinin-based combination therapy (ACT) only for positive results (i.e., ‘test and treat’). Whether the improvements in compliance with ‘test and treat’ guidelines are consistent across different malaria endemicity areas has not been examined.

**Methods:**

Data from 11 national, cross-sectional, outpatient malaria case-management surveys undertaken in Kenya from 2010 to 2016 were analysed. Four primary indicators (i.e., ‘test and treat’) and eight secondary indicators of artemether-lumefantrine (AL) dosing, dispensing, and counselling were measured. Mixed logistic regression models were used to analyse the annual trends in compliance with the indicators across the different malaria endemicity areas (i.e., from highest to lowest risk being lake endemic, coast endemic, highland epidemic, semi-arid seasonal transmission, and low risk).

**Results:**

Compliance with all four ‘test and treat’ indicators significantly increased in the area with the highest malaria risk (i.e., lake endemic) as follows: testing of febrile patients (OR = 1.71 annually; 95% CI = 1.51–1.93), AL treatment for test-positive patients (OR = 1.56; 95% CI = 1.26–1.92), no anti-malarial for test-negative patients (OR = 2.04; 95% CI = 1.65–2.54), and composite ‘test and treat’ compliance (OR = 1.80; 95% CI = 1.61–2.01). In the low risk areas, only compliance with test-negative results significantly increased (OR = 2.27; 95% CI = 1.61–3.19) while testing of febrile patients showed declining trends (OR = 0.89; 95% CI = 0.79–1.01). Administration of the first AL dose at the facility significantly increased in the areas of lake endemic (OR = 2.33; 95% CI = 1.76–3.10), coast endemic (OR = 5.02; 95% CI = 2.77–9.09) and semi-arid seasonal transmission (OR = 1.44; 95% CI = 1.02–2.04). In areas of the lowest risk of transmission and highland epidemic zone, none of the AL dosing, dispensing, and counselling tasks significantly changed over time.

**Conclusions:**

There is variability in health workers' compliance with outpatient malaria case-management guidelines across different malaria-risk areas in Kenya. Major improvements in areas of the highest risk have not been seen in low-risk areas. Interventions to improve practices should be targeted geographically.

## Background

Malaria continues to pose a huge public health threat in Africa [1]. In 2018, the WHO estimated 228 million cases and 405,000 deaths due to malaria, with over 90% of the deaths occurring in Africa [2]. Effective case-management remains the bedrock of malaria control [3]. In 2010, the globally recommended practice was universal testing of all suspected malaria patients and treatment of only test-positive patients with artemisinin-based combination therapy (ACT). This standard is referred to as ‘test and treat’ policy [4]. Effective supply chains must ensure universal and continuous availability of malaria testing and ACT as a basic pre-requisite for the policy implementation at health facilities. However, the effectiveness of test-based management also depends on how well healthcare workers comply with malaria case-management guidelines when attending to suspected cases [5-9].

Since 2010, despite the increased availability of parasitological diagnostics and ACT, studies among outpatients have reported sub-optimal health workers' compliance with ‘test and treat’ malaria guidelines [9-22], including poor ACT dosing, dispensing and counselling practices [10, 17, 22-24]. These studies have been undertaken on a small, often single facility sample [16, 19, 21, 23, 25-27]; at a single point of time [9, 10, 13, 15-17, 22, 23, 28]; have measured only a few indicators (e.g., treatment practices only) [14, 20, 25, 29, 30], or were limited to specific outpatient groups (e.g., children only) [12, 13, 19, 26, 28, 29, 31]. Several larger outpatient studies have suggested improvements in specific compliance indicators, such as testing of febrile patients or compliance with test-negative results [17, 22, 32, 33]. It has also been suggested that the malaria transmission setting influences ‘test and treat’ practices [34, 35], but trends in compliance indicators have not been widely explored in relation to malaria transmission intensities [19, 22].

In Kenya, health facility malaria case-management surveys have been regularly undertaken 11 times between 2010 and 2016 to monitor national progress in compliance with outpatient malaria guidelines. These surveys have shown improvements in a series of indicators since the launch of the ‘test and treat’ policy and surveys in 2010 [18, 21]. What have not been described previously are the temporal patterns of case-management compliance by health workers working in different malaria epidemiological settings. The present analysis aims to examine 2010–2016 compliance trends within and between malaria epidemiological zones in Kenya to identify sub-national deficiencies in compliance with guidelines.

## Methods

### Stratification of malaria epidemiological zones

**Fig. 1:**
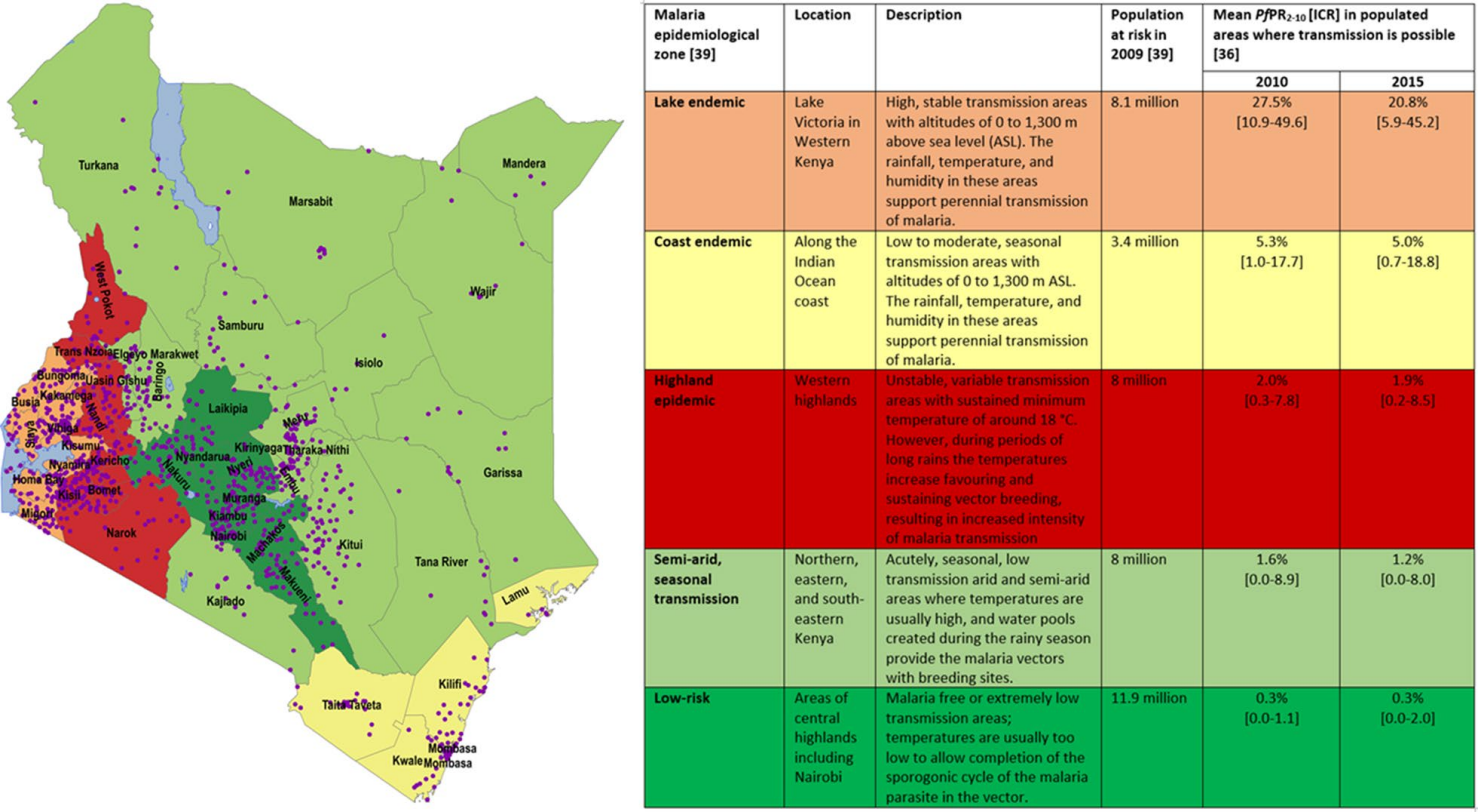
Malaria endemicity zones in Kenya and the sampled facilities as purple dots

**Fig. 2:**
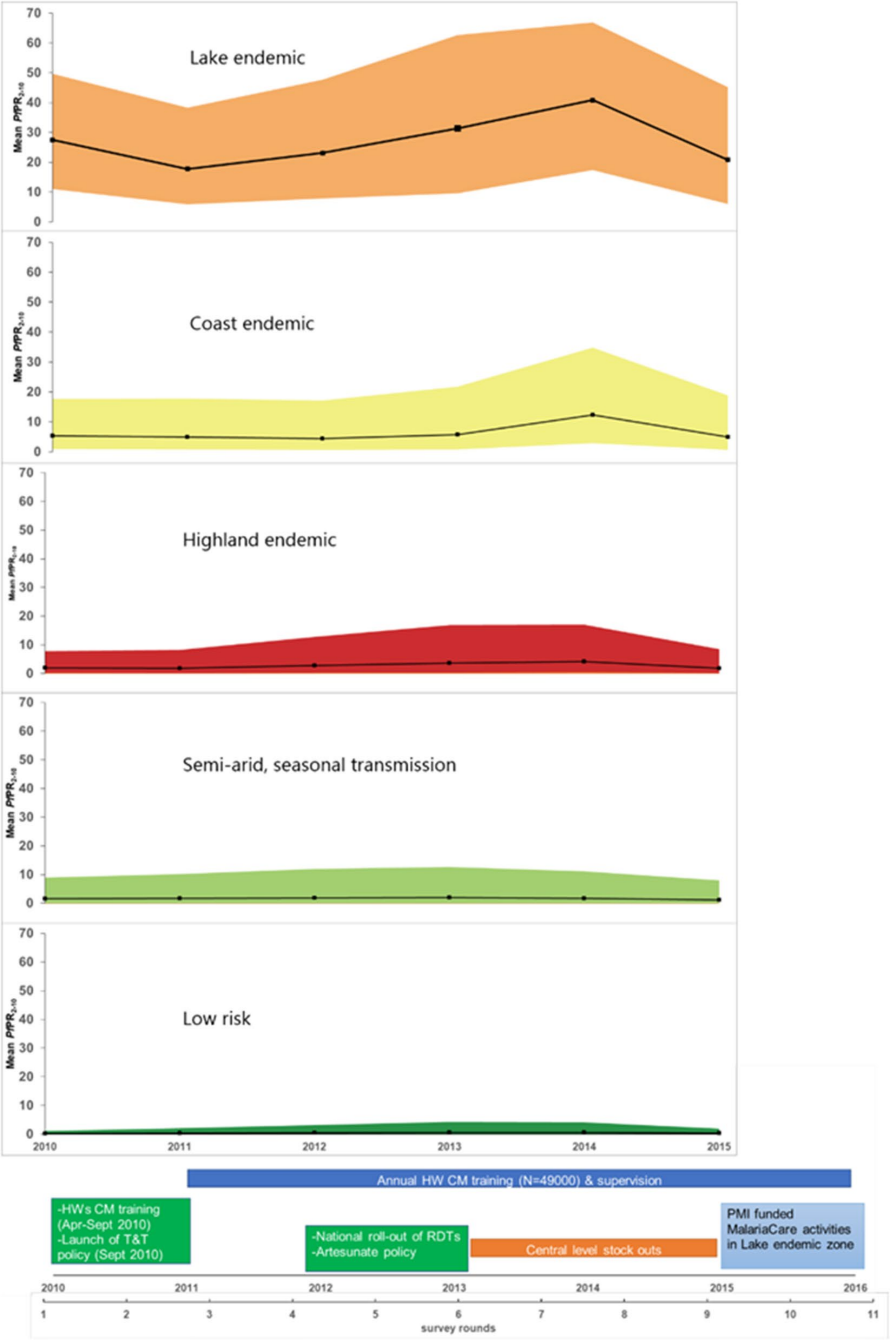
The annual mean *Pf*PR_2-10_ by malaria epidemiological zones and the test-based policy implementation activities, 2010–2016. To characterize the annual malaria parasite prevalence in the five malaria epidemiological zones of Kenya (lake endemic, coast endemic, highland epidemic, semi-arid seasonal transmission and low risk) we used previously published modelling work [36]. In brief, 5020 *Plasmodium falciparum* parasite prevalence (*Pf*PR) surveys at 3701 communities undertaken in Kenya between 1980 and 2015 were assembled. A spatio-temporal geostatistical model was fitted to predict the annual mean malaria risk and corresponding 2.5–97.5% interquartile credibility range (ICR) for children aged 2–10 years (*Pf*PR_2–10_) at 1 × 1 km spatial resolution. The model accounted for unmeasured spatio-temporal risk factors (structured random effects) and unexplained variation within communities (unstructured random effects) while standardizing for age. The annual average *Pf*PR_2–10_ and ICR by zone from 2010 to 2015 was then computed for populated areas where malaria transmission is possible. Areas that do not support malaria transmission were defined based on a temperature suitability index (TSI) (TSI zero areas) constructed using land surface temperatures, the average survival of Anopheles mosquitoes and the length of sporogony that must be completed within the lifetime of one Anopheline generation [41]. Populated areas were defined as locations with at least 1 person per km^2^ based on population density maps [42] available at Worldpop data geoportal [43]. The annual mean *Pf*PR_2-10_ in populated areas able to support transmission in each of the five MoH epidemiological zones (Fig. 1) were extracted and mapped using ArcMap 10.5 (ESRI Inc., Redlands, CA, USA) and shown for each year 2010–2015 in Fig. 2, against the major milestones of the policy change and implementation

Kenya supports a range of malaria transmission conditions within its national borders [36]. Despite major shifts in the intensity of malaria transmission over the last 25 years [36], the Kenya National Malaria Control Programme’s (NMCP) malaria stratification of the country considers five malaria epidemiological zones based on ecological differences and historical prevalence of malaria [37-40]. The five zones are as follows: (1) lake endemic: high transmission areas around Lake Victoria in western Kenya with stable malaria transmission all year round, described here as ‘high risk’; (2) coast endemic: low to moderate transmission areas along the Indian Ocean coast described here as ‘moderate risk’; (3) highland epidemic: these are areas of the western highlands with unstable, year-to-year variation in transmission; (4) semi-arid, seasonal transmission: arid and semi-arid areas of northern, eastern and south eastern Kenya with acute seasonal and low transmission; and, (5) low risk: areas of central highlands including Nairobi with low transmission [39, 40]. *Plasmodium falciparum* parasite prevalence standardized to ages 2–10 years (*Pf*PR_2-10_), a measure of malaria transmission, ranged between 0.3 and 28% in 2010, and 0.3 and 21% in 2015 across the five epidemiological zones [36] (Fig. 1). The annual trends in *Pf*PR_2-10_ across the five epidemiological zones between 2010 and 2015 are presented in Fig. 2. The population at risk ranged between 3 and 12 million people across the five zones in 2009 (Fig. 1) [39].

## Malaria case-management standards

**Fig. 3:**
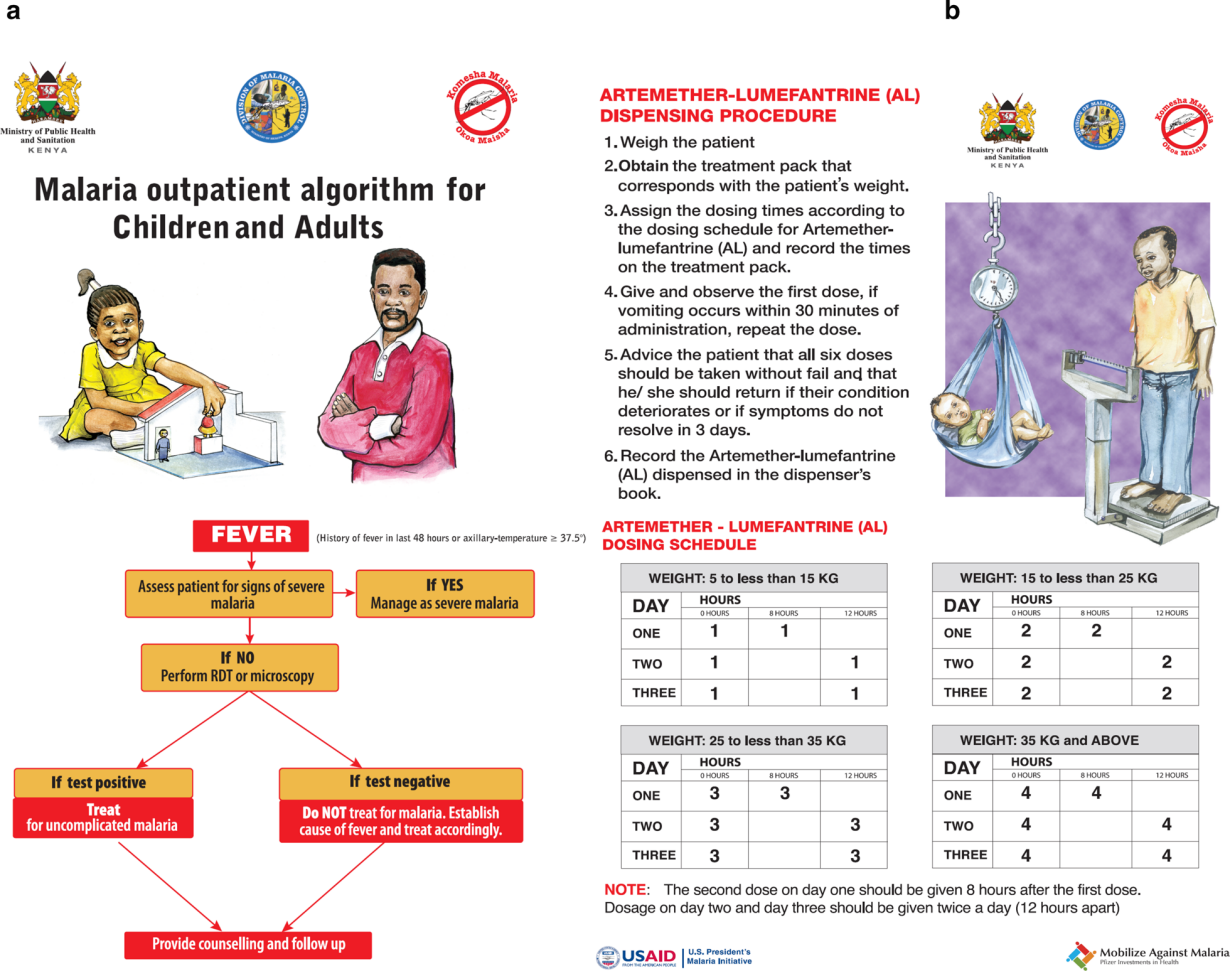
Outpatient malaria case-management recommendation algorithm

Figure 3a and b are the 2010 outpatient malaria case-management algorithms that apply to all epidemiological zones, age groups and levels of care in Kenya. With respect to malaria diagnosis, the 2010 policy recommends universal parasitological testing of all patients with fever across all areas of malaria transmission with either malaria microscopy or rapid diagnostic tests (RDTs), and subsequent anti-malarial treatment for test-positive patients only [37]. The recommended first-line treatment for uncomplicated malaria is artemether–lumefantrine (AL) since 2006 [44, 45]. Finally, recognizing the greater complexity of AL dosing and administration compared to the previous single-dose monotherapy, the 2010 guidelines for health workers emphasized AL dosing, dispensing and counselling tasks that should be performed for all patients treated with AL (Fig. 3b) [37]. Since the launch of new case-management policy in 2010, its implementation has been countrywide, across all epidemiological zones, achieved through routine programmatic activities such as strengthening of the supply chain for ‘test and treat’ commodities, dissemination of revised guidelines and job aids, a series of in-service case-management training for health workers and strengthening of the supportive supervision and quality assurance for malaria microscopy.

Other contextual factors during this period include central level stock-outs of AL and RDTs in 2013 and 2014 due to a fire at the Kenya Medical Supplies Agency (KEMSA) stores and levy tax that delayed the delivery of commodities. Moreover, in 2015, MalariaCare, a USAID partnership, began working in the lake endemic zone to improve the quality of diagnosis and treatment of malaria and other febrile illnesses [46]. The implemented activities included case-management and laboratory training, on-site supervision, and mentoring [known as ‘outreach training and support supervision’ (OTSS)], electronic data collection and follow-up evaluation, and implementation of the lessons learned. By 2016, two rounds of OTSS were conducted reaching 98% coverage of the facilities in the region and this might have contributed to the high compliance levels noted in the zone at the last survey (Fig. 2) [47, 48].

### Data sources

The study utilized data from 11 national, biannual, cross-sectional, cluster sample health facility surveys undertaken between January 2010 (before the ‘test and treat’ policy was introduced) and July 2016. For each survey, a proportionate stratified random sample of facilities was drawn from the Ministry of Health (MoH) master list of all public health facilities, taking into consideration administrative boundaries to ensure national representativeness [49]. The number of assessed facilities ranged between 169 and 176 facilities per survey. At each of the surveyed facilities, data collection methods included health facility assessments, interviews with health workers and exit interviews with all eligible outpatients during one survey day when they were ready to leave the facility [18, 21]. The patients’ exit interviews included all non-referred for admission and non-pregnant patients weighing > 5 kg across all age groups and presenting for an initial visit with fever or history of fever. Information was collected from patient-held cards about malaria tests requested, test results, treatment prescribed, and direct questioning about drug dispensing and counselling practices during the facility visit. At each facility, the availability of AL, RDTs and malaria microscopy was determined for the survey day. Data quality was assured through five days of training of the field workers, double-entry into Microsoft Access database, and data files comparisons using a verification program in Microsoft Access and referring to paper-based questionnaires.

### Indicators, analytical approaches, and statistical analysis

A set of 12 indicators of health workers' compliance with national malaria case-management guidelines was selected (Table 1). Four primary indicators reflected health worker decisions to test febrile patients for malaria, comply respectively with test-positive and test-negative results, and a composite indicator of ‘test and treat’ compliance among all febrile patients. Eight secondary indicators included compliance with the recommended AL dosing among patients who had AL prescribed with complete dosage prescriptions, and seven AL dispensing and counselling tasks evaluated among patients who had AL prescribed and dispensed to be taken at home (Table 1). Since the lack of commodities precludes measurements of ‘test and treat’ compliance, the analysis was restricted to the facilities with available AL and malaria diagnostics.

For each of the 12 compliance indicators, the primary analytic approach was to estimate temporal trends within each malaria risk zone and compare the trends across the zones over 11 survey rounds from January 2010 to July 2016. For a contextual understanding of these trends, compliance levels for each indicator stratified by malaria risk are calculated for the baseline and the last survey. For each indicator, multilevel mixed-effects logistic regression model was fitted. The clustering of patients within health facilities was accounted for by fitting a random intercept for each health facility [50]. The temporal trends were examined by fitting time as a continuous variable and interaction term between each malaria risk zone and time. Tests for significant interactions were conducted using the Wald test. For the baseline survey, conducted in January 2010, time was set to zero and time in months since the baseline survey was calculated for the consecutive surveys. This was then converted to years by dividing by 12 to estimate the annual change in the performance of each indicator. P-values less than 0.05 were considered statistically significant. The results are presented as time trends (annual odds ratios) with 95% confidence intervals (CI) and plots of the observed proportion of patients with the outcome *versus* predicted marginal regression lines. The area under the receiver operating characteristic (ROC) curves was used to evaluate the goodness of fit of the final models. All analyses were conducted using Stata version 14 (StataCorp, College Station, TX, USA).

## Results

**Fig. 4:**
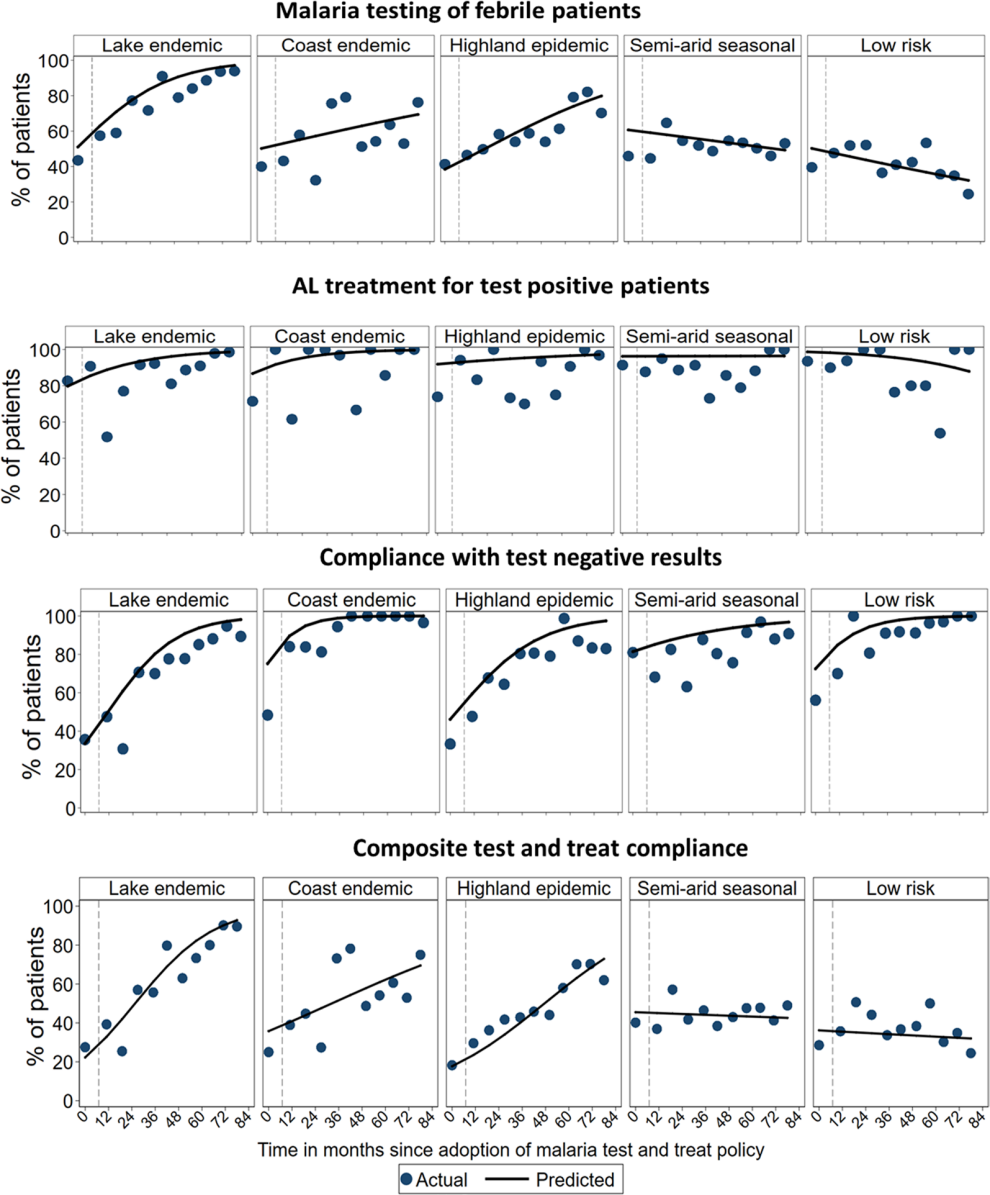
Time trends in health workers' compliance with outpatient malaria ‘test and treat’ policy by malaria epidemiological zones

**Fig. 5:**
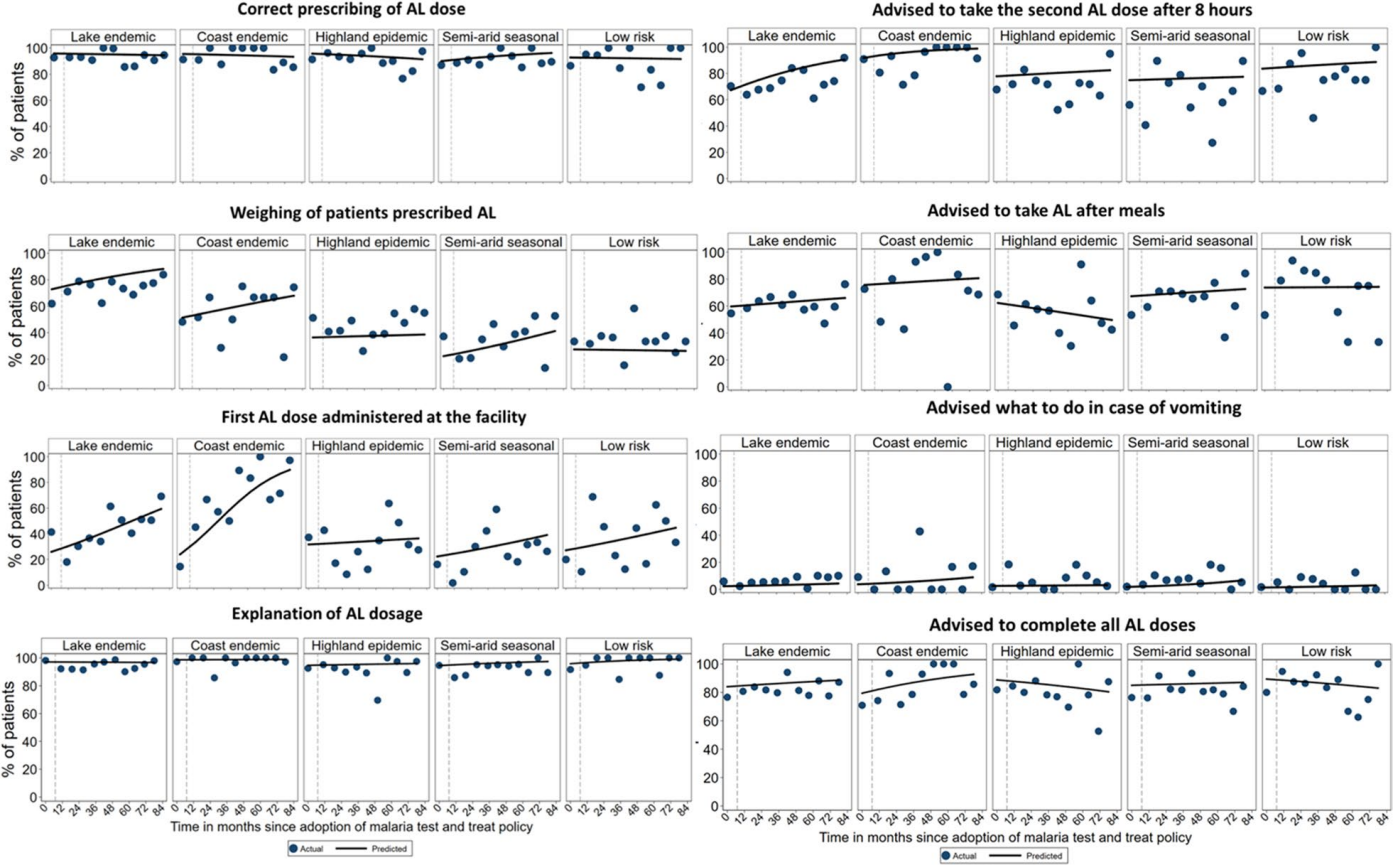
Time trends in health workers’ compliance with AL dosing, dispensing, and counselling by malaria endemicity

The trend analysis across five epidemiological zones included a range of 932 to 2,766 febrile patients aged between 1 month and 98 years, 129 to 1,178 test positive, 321 to 882 test negative, 191 to 1,620 AL prescribed and 184 to 1,570 patients who had AL dispensed at facilities with available ‘test and treat’ commodities across 11 survey rounds conducted from 2010 to 2016. Over the 11 survey rounds, the number of facilities and health workers who saw febrile patients across the five zones ranged between 93 and 331 (Fig. 1) and 126 and 408, respectively. Table 1 shows the proportions of patients for whom health workers complied with malaria guidelines at the baseline and the last survey, while Table 2, and Figs. 4 and 5 show the compliance trends within each of the five malaria risk zones across all survey rounds.

**Table 1:** Compliance with the outpatient malaria guidelines by epidemiological zones in 2010 and 2016, Kenya

Indicators of malaria case-management quality	Lake endemic	Coast endemic	Highland epidemic	Semi-arid, seasonal transmission	Low risk
Primary indicators—‘test and treat’ compliance					
Malaria testing of febrile patients					
Baseline-2010	43.5 (177/407)	40.0 (80/200)	41.4 (86/208)	45.9 (112/244)	39.6 (72/182)
Last survey-2016	93.9 (216/230)	76.3 (61/80)	70.3 (85/121)	53.1 (77/145)	24.5 (24/98)
AL treatment for test positive patients					
Baseline	82.7 (86/104)	71.4 (35/49)	73.9 (17/23)	91.4 (64/70)	93.6 (29/31)
Last survey	98.6 (139/141)	100 (32/32)	96.9 (31/32)	100 (12/12)	100 (3/3)
Compliance with test negative results					
Baseline	35.6 (26/73)	48.4 (15/31)	33.3 (21/63)	81.0 (34/42)	56.1 (23/41)
Last survey	89.3 (67/75)	96.6 (28/29)	83.0 (44/53)	90.8 (59/65)	100 (21/21)
Composite “test and treat” compliance					
Baseline	27.5 (112/407)	25.0 (50/200)	18.3 (38/208)	40.2 (98/244)	28.6 (52/182)
Last survey	89.6 (206/230)	75.0 (60/80)	62.0 (75/121)	49.0 (71/145)	24.5 (24/98)
Secondary indicators—AL dosing, dispensing and counselling compliance					
Correct prescribing of AL dose					
Baseline	92.7 (240/259)	91.2 (83/91)	91.3 (105/115)	86.9 (106/122)	86.4 (51/59)
Last survey	94.6 (139/147)	85.3 (29/34)	97.5 (39/40)	89.5 (17/19)	100 (3/3)
Weighing of patients prescribed AL					
Baseline	61.9 (169/273)	48.2 (53/110)	51.2 (62/121)	37.2 (55/148)	33.3 (20/60)
Last survey	83.9 (125/149)	74.3 (26/35)	55.0 (22/40)	52.6 (10/19)	33.3 (1/3)
First AL dose administered at the facility					
Baseline	41.4 (113/273)	14.6 (16/110)	37.2 (45/121)	16.2 (24/148)	20.0 (12/60)
Last survey	69.1 (103/149)	97.1 (34/35)	27.5 (11/40)	26.3 (5/19)	33.3 (1/3)
Explanation of AL dosage					
Baseline	98.2 (268/273)	97.3 (107/110)	92.6 (112/121)	94.6 (140/148)	91.7 (55/60)
Last survey	98.0 (146/149)	97.1 (34/35)	97.5 (39/40)	89.5 (17/19)	100 (3/3)
Advised to take the second AL dose after 8 h					
Baseline	70.3 (192/273)	90.9 (100/110)	67.8 (82/121)	56.1 (83/148)	66.7 (40/60)
Last survey	91.9 (136/148)	91.4 (32/35)	95.0 (38/40)	89.5 (17/19)	100 (3/3)
Advised to take drugs after meals					
Baseline	54.6 (149/273)	72.7 (80/110)	68.6 (83/121)	53.4 (79/148)	53.3 (32/60)
Last survey	76.2 (112/147)	68.6 (24/35)	42.5 (17/40)	84.2 (16/19)	33.3 (1/3)
Advised what to do in case of vomiting					
Baseline	5.9 (16/273)	9.1 (10/110)	1.7 (2/121)	2.0 (3/148)	1.7 (1/60)
Last survey	10.1 (15/149)	17.1 (6/35)	2.5 (1/40)	5.3 (1/19)	0 (0/3)
Advised to complete all AL doses					
Baseline	76.6 (209/273)	70.9 (78/110)	81.8 (99/121)	76.4 (113/148)	80.0 (48/60)
Last survey	87.2 (129/148)	85.7 (30/35)	87.5 (35/40)	84.2 (16/19)	100 (3/3)

## Malaria ‘test and treat’ compliance

The composite compliance with the ‘test and treat’ policy, measured as a febrile patient tested for malaria and treated according to the test result, was low at the baseline and ranged from 18 to 40% across the five zones (Table 1). Within three epidemiological zones, the odds of compliance increased significantly with an annual change of 80% in the lake endemic (OR = 1.80 annually; 95% CI = 1.61–2.01), 47% in the highland epidemic (OR = 1.47; 95% CI = 1.32–1.65) and 24% in the coast endemic (OR = 1.24; 95% CI = 1.05–1.46) zones. The annual trends were significantly higher within the lake endemic compared to the highland epidemic (p = 0.015) and coast endemic (p < 0.001) zones. There were no significant changes in the performance trends within the semi-arid seasonal transmission and low risk zones (Table 2 and Fig. 4). At the last survey, the composite ‘test and treat’ performance was highly variable across the zones and ranged from 25% in the low risk area to 90% in the lake endemic zone (Table 1).

**Table 2:** Time trends in health workers’ compliance with the outpatient malaria guidelines by epidemiological zones, 2010–2016

	Lake endemic		Coast endemic		Highland epidemic		Semi-arid, seasonal transmission		Low risk	
	N (Mean^†^ %)	Time trend^*^ [95% CI] (p-value)	N (Mean %)	Time trend [95% CI] (p-value)	N (Mean %)	Time trend [95% CI] (p-value)	N (Mean %)	Time trend [95% CI] (p-value)	N (Mean %)	Time trend [95% CI] (p-value)
Primary indicators—‘test and treat’ compliance										
Malaria testing of febrile patients	2766 (74.5)	*1.71 [1.51–1.93] (< 0.001)*	932 (54.6)	1.13 [0.95–1.35] (0.165)	1898 (58.5)	*1.32 [1.18–1.49] (< 0.001)*	2071 (51.2)	0.93 [0.83–1.04] (0.212)	1506 (41.0)	0.89 [0.79–1.01] (0.064)
AL treatment for test positive patients	1178 (88.4)	*1.56 [1.26–1.92] (< 0.001)*	189 (87.8)	*1.77 [1.07–2.94] (0.026)*	304 (86.2)	1.18 [0.85–1.66] (0.326)	400 (88.8)	1.01 [0.72–1.40] (0.973)	129 (87.6)	0.70 [0.44–1.12] (0.134)
Compliance with test negative results	882 (71.5)	*2.04 [1.65–2.54] (< 0.001)*	321 (90.7)	*3.12 [1.76–5.53] (< 0.001)*	806 (74.8)	*1.80 [1.45–2.24] (< 0.001)*	661 (83.2)	*1.34 [1.06–1.69] (0.013)*	490 (88.0)	*2.27 [1.61–3.19] (< 0.001)*
Composite “test and treat” complianc**e**	2766 (60.5)	*1.80 [1.61–2.01] (< 0.001)*	932 (48.9)	*1.24 [1.05–1.46] (0.010)*	1898 (45.6)	*1.47 [1.32–1.65] (< 0.001)*	2071 (43.7)	0.98 [0.88–1.09] (0.726)	1506 (36.1)	0.97 [0.87–1.09] (0.651)
Secondary indicators—AL dosing, dispensing, and counselling compliance										
Correct prescribing of AL dose	1561 (93.5)	0.95 [0.85–1.08] (0.446)	251 (92.4)	0.94 [0.74–1.20] (0.617)	637 (91.8)	0.89 [0.76–1.06] (0.192)	687 (90.5)	1.17 [0.97–1.41] (0.107)	187 (90.4)	0.98 [0.73–1.31] (0.880)
Weighing of patients prescribed AL	1570 (72.5)	*1.17 [1.01–1.36] (0.044)*	269 (55.0)	1.11 [0.83–1.49] (0.468)	635 (44.7)	1.02 [0.84–1.23] (0.874)	686 (33.8)	1.15 [0.93–1.42] (0.204)	184 (35.9)	0.99 [0.73–1.35] (0.960)
First AL dose administered at the facility	1570 (44.6)	*2.33 [1.76–3.10] (< 0.001)*	269 (49.1)	*5.02 [2.77–9.09] (< 0.001)*	635 (30.9)	1.25 [0.90–1.75] (0.191)	686 (23.8)	*1.44 [1.02–2.04] (0.039)*	184 (29.4)	1.47 [0.85–2.53] (0.168)
Explanation of AL dosage	1569 (95.1)	0.98 [0.84–1.14] (0.794)	269 (97.8)	1.04 [0.69–1.56] (0.869)	635 (92.6)	1.04 [0.85–1.28] (0.684)	686 (92.6)	1.13 [0.90–1.43] (0.297)	184 (95.1)	1.28 [0.79–2.05] (0.317)
Advised to take the second AL dose after 8 h	1569 (74.0)	*1.27 [1.09–1.48] (0.003)*	269 (90.3)	1.37 [0.93–2.02] (0.108)	635 (71.2)	1.05 [0.86–1.27] (0.656)	686 (62.2)	1.02 [0.83–1.27] (0.838)	184 (73.9)	1.07 [0.77–1.49] (0.673)
Advised to take drugs after meals	1568 (61.2)	1.04 [0.95–1.14] (0.364)	269 (72.5)	1.05 [0.87–1.26] (0.643)	635 (55.4)	0.92 [0.82–1.05] (0.209)	686 (63.6)	1.04 [0.90–1.20] (0.572)	184 (69.6)	1.00 [0.80–1.26] (0.975)
Advised what to do in case of vomiting	1570 (6.1)	1.10 [0.94–1.29] (0.225)	269 (11.5)	1.15 [0.87–1.53] (0.325)	635 (6.3)	1.03 [0.82–1.30] (0.800)	686 (5.8)	1.23 [0.96–1.59] (0.107)	184 (3.8)	1.13 [0.70–1.82] (0.612)
Advised to complete all AL doses	1567 (83.0)	1.06 [0.96–1.18] (0.258)	269 (79.2)	1.20 [0.97–1.49] (0.090)	635 (80.8)	0.91 [0.79–1.04] (0.165)	686 (81.0)	1.03 [0.88–1.20] (0.743)	184 (83.7)	0.92 [0.71–1.19] (0.521)

^**†**^The mean indicator value across 11 survey rounds;

^*^Change in the odds of the indicator per year within each malaria risk zone; Bold font indicates a significant trend at p < 0.05

The trends in the specific components of the ‘test and treat’ pathway are specified below. The levels of compliance with malaria testing of febrile patients were similar across the five zones and ranged between 40 and 46% at the baseline (Table 1). The proportion of patients who were tested significantly increased over time in the lake endemic to 94% (OR = 1.71; 95% CI = 1.51–1.93) and highland epidemic areas to 70% (OR = 1.32; 95% CI = 1.18–1.49) (Table 2). The annual increase in testing was significantly higher in the lake endemic compared to the highland epidemic areas (p = 0.003). In contrast, there was some evidence of a decline in the proportion of patients tested over time within the low-risk zone to 25% by 2016 (OR = 0.89; 95% CI = 0.79–1.01). No significant changes were observed in the semi-arid seasonal transmission and coast endemic zones (Table 2). In the coast endemic zone, variability in the proportion of tested patients was observed between surveys resulting in no consistent time trend (Table 2 and Fig. 4).

Prescription of AL to confirmed malaria cases ranged between zones from 71% in the coast endemic area to 94% in the low risk zone at baseline (Table 1). Despite relatively high baseline levels, AL prescribing for test-positive patients showed significant and similar (p = 0.638) time trends within the lake (OR = 1.56; 95% CI = 1.26–1.92) and coast endemic (OR = 1.77; 95% CI = 1.07–2.94) zones and no significant changes within the highland epidemic, semi-arid seasonal transmission, and low risk zones over time (Table 2 and Fig. 4). In the last survey, a range of 97 to 100% of confirmed malaria cases were prescribed AL across the five zones (Table 1).

Lastly, withholding anti-malarial treatment for patients who tested negative was variable at the baseline and ranged from 33 to 81% across the five zones. The semi-arid seasonal transmission areas had the highest levels of compliance (81%) while 33 and 36% of test-negative patients were not, respectively, prescribed an anti-malarial in the highland and lake endemic zones (Table 1). Across all five malaria risk zones, compliance with test-negative results significantly increased over time with an annual increase in the odds of thrice within the coast endemic (OR = 3.12; 95% CI = 1.76–5.53), twice in the low risk (OR = 2.27; 95% CI = 1.61–3.19) and lake endemic (OR = 2.04; 95% CI = 1.65–2.54), 80% in the highland epidemic (OR = 1.80; 95% CI = 1.45–2.24) and 34% in the semi-arid seasonal transmission (OR = 1.34; 95% CI = 1.06–1.69) areas. The annual trends were significantly higher within the lake endemic (p = 0.008), coast endemic (p = 0.007), low risk (p = 0.012) compared to the semi-arid seasonal transmission areas (Table 2 and Fig. 4). During the last survey, the proportion of test-negative patients who were not prescribed an anti-malarial was high in all zones and ranged from 83% in the highland epidemic zone to 100% in the low risk area (Table 1).

### AL dosing, dispensing, and counselling compliance

At the baseline, the levels of eight AL dosing, dispensing, and counselling performance tasks differed between the evaluated tasks (Table 1). Correct prescribing of AL dose and explaining of AL dose to be taken at home was at high-performance levels and for the respective tasks ranged across five zones from 86 to 93% and from 92 to 98%. The health workers’ performance of the remaining six tasks was much lower at the baseline with the following task range across malaria zones: weighing 33–62%; administration of the first AL dose 15–41%; and, provision of advice on taking the second AL dose after eight hours 56–91%, to take AL after meals 53–73%, to complete all AL doses 71–82%, and what to do in case of vomiting 2–9% (Table 1).

The proportion of patients who had the first dose of AL administered at the facility significantly increased over time in three malaria zones with the odds increasing five times annually within the coast endemic (OR = 5.02; 95% CI = 2.77–9.09), twice in the lake endemic (OR = 2.33; 95% CI = 1.76–3.10) and 44% within the semi-arid seasonal transmission (OR = 1.44; 95% CI = 1.02–2.04) zones. The annual trends were significantly higher within the coast endemic zone compared to lake endemic (p = 0.014) and semi-arid seasonal transmission areas (p < 0.001). In the lake endemic zone, significant annual improvement trends were also observed in the weighing of patients (OR = 1.17; 95% CI = 1.01–1.36) and in advising patients to take the second AL dose after eight hours (OR = 1.27; 95% CI = 1.09–1.48). In the highland and low risk areas, none of the eight monitored tasks showed significant changes over time (Table 2 and Fig. 5).

Proportions of patients who received correct AL dosing, explanation of AL dose and provision of advice to take AL after meals, to complete all AL doses, and what to do in case of vomiting showed no significant changes over time in any of the five epidemiological zones (Table 2 and Fig. 5). With the exception of advice on vomiting, which was rarely offered throughout the monitoring period and across all zones (range 0 to 17%), the last survey found that all of the seven remaining AL dosing, dispensing and counselling tasks were performed for more than two-thirds of the patients in the lake and coast endemic areas, while in the highland, semi-arid seasonal transmission and low risk areas, the performance in the administration of the first AL dose at the facility was significantly lower and ranged across these three zones from 26 to 33% (Table 1).

## Discussion

The analysis across malaria epidemiological zones revealed important spatial differences in health workers' compliance with outpatient guidelines, which has implications for future malaria case-management in Kenya. Overall, major improvement trends in health workers’ ‘test and treat’ practices were found in an area of the highest malaria risk around Lake Victoria where by the end of the monitoring period over 90% of all febrile patients were both tested and treated according to guidelines. Moreover, it was only in this area that the performance of several drug dispensing and counselling tasks significantly improved. In low malaria risk areas, and particularly in areas of very low risk in central Kenya, no significant changes have been observed in the performance of any of the case-management tasks, except in compliance with the no anti-malarial policy for test-negative, patients which improved in all epidemiological zones.

Malaria testing of all febrile patients is a critical, early step of malaria case-management [35] which if not systematically performed leads to missed malaria diagnosis both in high and low transmission settings [51-54]. Low malaria testing rates of fevers have been commonly reported as one of the weakest components of outpatient malaria case-management despite the testing readiness at health facilities [10, 16, 22, 26, 55, 56]. The 2010 findings showing less than half of febrile patients tested for malaria at facilities with available ‘test and treat’ commodities concur with these reports. Notable differences in testing trends between high and low malaria risk areas were observed between 2010 and 2016. Major improvements in Kenyan high malaria risk areas reaching over 90% of tested febrile patients, though imperfect, are encouraging findings. However, they are in stark contrast with low risk areas where no improvement, or even declining trends resulting in only a quarter of febrile patients tested in areas of the lowest risk were observed. Both behaviours have been commonly attributed to health workers’ practices considering pre-test probability perceptions of malaria [34], as similarly shown for other diseases where local epidemiology of diseases influence health workers’ selection of diagnostic tests [57, 58]. The undertesting in low risk areas does not only compromise case-management but also prospects of ensuring good quality routine data through the District Health Information Software2 (DHIS2) for surveillance of parasitologically confirmed cases as an intervention for malaria elimination to which Kenya is aspiring in these areas [59, 60]. Carefully designed and evaluated interventions changing health workers’ testing behaviour in low malaria risk areas should be a case-management priority of malaria control programmes and operational researches.

Health workers’ compliance with test-negative results is an important case-management component determining the cost–benefit of the test and treat policy for malaria [61]. Despite the improvement in the trends in this practice across Africa [32], rates of 20–30% of test-negative outpatients treated for malaria have been estimated with large variability of non-compliant practices between individual studies [62]. With respect to malaria transmission, malaria treatments for test-negative patients have been reported in 57 and 7% of high and low malaria risk hospital outpatients, respectively, in western Kenya in 2012/2013 [19]. This analysis showed high levels of non-compliant practices in 2010 with subsequent major improvements in all epidemiological zones resulting in nearly all test-negative patients not treated for malaria in low risk areas but also in 90% of the patients for whom health workers complied with the guidelines in areas of the highest malaria risk around the Lake Victoria in 2016. In these high malaria risk areas, likely due to the highest prevalence of disease and a long history of presumptive treatment practices [45], the behavioural changes were the slowest and, even though they are imperfect, high levels of compliance with test-negative results have been observed five years after the policy change.

Treatment with highly efficacious ACT for confirmed cases is one of the direct measures of case-management effectiveness [6, 7]. In Kenya, the ACT policy recommending AL for uncomplicated malaria was implemented in 2006 [44] and health workers’ prescribing preferences for other therapy in the presence of ACT has been common during early years of the policy implementation [63, 64], as is similarly shown in other African countries [65-68]. Such practices, though at lower levels, have persisted until 2010 in Kenya and were particularly pronounced in the high-risk lake endemic and coastal areas where the use of parenteral anti-malarials on an outpatient basis was a frequent treatment practice [19, 21]. From 2010 onwards, significant improvements were observed in these areas and during the surveys in 2016 irrational use of injectable anti-malarials has nearly disappeared and AL prescribing for confirmed cases has been standardized across all areas of malaria risk.

Despite modest changes in the overall quality of AL dispensing and counselling across the zones, the major improvement trends observed in the administration of the first AL dose at the facility in the high-risk lake endemic (OR = 2.3) and coastal areas (OR = 5.0) should not be underestimated. Administration of the first AL dose ensures prompt treatment for malaria and is one of the main factors determining patients’ adherence to medicines and treatment effectiveness [69-71]. A series of outpatient evaluations, including interventional studies, has not only reported sub-optimal performance of this task but also resilience to practice change [10, 17, 22]. Under routine conditions of the policy implementation, major improvements have been seen in high transmission areas where most ACT is prescribed in Kenya, despite a potential practice conflict between prompt treatment and recommended AL dosing after a meal. Alongside improvements in the AL administration at the facility, declining trends in advising patients to take AL at home after a meal were not observed. Interestingly, in areas of low malaria risk, where anti-malarials are rarely prescribed, only minor improvement trends have been observed and AL administration in these areas remained at very low levels. The findings imply that in low risk areas high workload may not be a reason for poor dispensing practices for anti-malarials, as commonly reported in the past [9, 17, 33, 72]. Finally, across the zones and over the years, most patients were correctly dosed, advised on dosing at home and told to “finish” all doses, however, advice on what do in case of vomiting was rarely provided. Since vomiting is common and non-replacement of doses compromises patients’ adherence and treatment outcomes [73], further investigations to understand such practices are required. Accountability for replacement doses at the facilities might be one of the systems factors worth exploring as a reason for the low levels of advising patients on what to do in case of vomiting.

This study has some limitations. Within some zones, there was a possibility of failure to detect significant trends due to small sample sizes for some indicators and the presence of variability in compliance levels across the years. Second, the impact of diagnostic and treatment practices on true malaria cases among the universe of febrile patients could not be established due to lack of gold standard testing which was beyond the scope of this compliance study. Finally, the focus of analyses in this manuscript is compliance trends across five epidemiological zones without adjusting for other factors, the potential correlations between compliance and other interventional and non-interventional factors will be explored separately.

## Conclusion

Trends in health workers’ compliance with outpatient malaria case-management guidelines differed across different malaria epidemiological zones between 2010 and 2016 in Kenya. The major improvements in health workers’ ‘test and treat’ practices, including improved performance of drug dispensing and counselling, were observed in areas of the highest malaria risk around Lake Victoria. Conversely, in low risk areas, no significant changes have been seen in the performance of any of the case-management tasks, except in compliance with test-negative results, which indeed increased across all epidemiological zones. By the end of 2016, anti-malarial treatment compliance with test-positive results, and somewhat less to test-negative results, has largely become the standard practice across all zones while major undertesting gaps of febrile patients have been revealed in areas of low risk, calling for interventions to change health workers practices in these areas.

## Data Availability

The datasets used and analysed during the current study are available from the corresponding author on reasonable request.
